# Spatial capture–recapture analysis of artificial cover board survey data reveals small scale spatial variation in slow-worm *Anguis fragilis* density

**DOI:** 10.1098/rsos.170374

**Published:** 2017-09-13

**Authors:** Benedikt R. Schmidt, Anita Meier, Chris Sutherland, J. Andy Royle

**Affiliations:** 1 Info Fauna Karch, Passage Maximilien-de-Meuron 6, 2000 Neuchâtel, Switzerland; 2 Institut für Evolutionsbiologie und Umweltwissenschaften, Universität Zürich, Winterthurerstrasse 190, 8057 Zürich, Switzerland; 3 Zürcher Hochschule für Angewandte Wissenschaften, Grüental, 8820 Wädenswil, Switzerland; 4 Department of Environmental Conservation, University of Massachusetts – Amherst, Amherst, MA, USA; 5 U.S. Geological Survey, Patuxent Wildlife Research Center, Laurel, MD, USA

**Keywords:** abundance, reptile, artificial cover object, home range, spatial capture–recapture, translocation

## Abstract

Vague and/or ad hoc definitions of the area sampled in monitoring efforts are common, and estimates of ecological state variables (e.g. distribution and abundance) can be sensitive to such specifications. The uncertainty in population metrics due to data deficiencies, vague definitions of space and lack of standardized protocols is a major challenge for monitoring, managing and conserving amphibian and reptile populations globally. This is especially true for the slow-worm (*Anguis fragilis*), a cryptic and fossorial legless lizard; uncertainty about spatial variation in density has hindered conservation efforts (e.g. in translocation projects). Spatial capture–recapture (SCR) methods can be used to estimate density while simultaneously and explicitly accounting for space and individual movement. We use SCR to analyse mark–recapture data of the slow-worm that were collected using artificial cover objects (ACO). Detectability varied among ACO grids and through the season. Estimates of slow-worm density varied across ACO grids (13, 45 and 46 individuals ha^−1^, respectively). The estimated 95% home range size of slow-worms was 0.38 ha. Our estimates provide valuable information about slow-worm spatial ecology that can be used to inform future conservation management.

## 1. Introduction

As we have entered the sixth mass extinction [1], conservation biologists need standardized tools that allow them to describe the state of populations so that declines can be quantified and management actions and interventions can be rigorously evaluated [2]. Reliable estimates of the distribution and abundance of species are of fundamental importance for basic and applied ecology. Yet, despite their importance, neither abundance nor distribution have an intrinsic definition as they are derived quantities of a point pattern. The point pattern describes the number and the location of individuals of a species in space, so to quantify abundance and distribution, one must first define a unit area. Abundance is then defined as the number of individuals within that unit area and distribution is the case where abundance is dichotomized into presence and absence. Distribution and abundance will not be the same for different spatial units (e.g. 1 hectare, 1 km^2^, etc.; [3–5]). While conceptually straightforward, a major criticism of many approaches to-date is that space is not explicitly incorporated into models of abundance and distribution, and thus inference about ecological state variables such as abundance and distribution patterns is either non-spatial, or space is loosely defined and may be biased by an inaccurate definition of the effective sampling area [6].

A clear definition of space is important for two reasons. First, abundance is often converted to density by dividing the abundance estimate by the study area. For populations living in continuous habitat, the area used by individuals is not known and therefore the spatial extent of the study area is often not well defined [7]. Second, home ranges of individuals overlap traps or sampling devices to variable extents. This may lead to heterogeneous individual detection probabilities, and consequently to biased estimates of abundance [6,8,9]. In many monitoring studies, abundance (population size or density) is used to describe the state of populations. Mark–recapture methods are commonly used to estimate abundance, but because defining the spatial extent of the sampled population is difficult, space is often not taken explicitly into account [6,10]. For some species, space is well defined, e.g. for pond-breeding amphibians where the pond is usually the spatial unit. For other species, however, space is more challenging to define because there are no obvious natural boundaries. For example, the Swiss breeding bird survey uses arbitrary 1 km^2^ units [11] but birds that are counted may inhabit areas beyond the sample unit boundary.

Spatial capture–recapture methods (SCR; [12–14]) use data on the location of individual encounters to accommodate both the spatial structure of populations (i.e. how individuals are distributed in space), and the spatial structure of population sampling. In doing so, they can resolve technical limitations of ordinary CR methods such as unknown sample area and induced detection heterogeneity, but also they can incorporate realistic biological structure into capture–recapture models such as landscape connectivity [15,16] or spatial variation in density [13]. SCR can be easily applied to data collected using any sampling design for which sample locations and individual identities are known [17,18].

There are a range of field methods used in mark–recapture studies where it would be straightforward to formally incorporate space through the use of SCR. For example, for many ground-dwelling species, the use of artificial cover objects (ACO) has proven to be a useful method to collect mark–recapture data [19,20]. Hesed [21] listed some of the advantages of ACO: they require a relatively small investment of time and resources to establish and maintain, induce little risk to the animals being monitored, result in low levels of disturbance to habitats, and allow cover objects to be standardized in number and size (although the latter may sometimes be difficult if natural cover objects are present; [22]). Importantly, surveying amphibians and reptiles using ACO requires relatively limited training and can therefore be used in citizen science monitoring programmes (such as National Amphibian and Reptile Recording Scheme in the United Kingdom [22]). Although the use of ACO can reduce between-observer variability, ACO surveys can have very high spatial and temporal variation in capture rates (e.g. because of heterogeneity in soil moisture or pH) [23].

We use data from a spatial capture–recapture study on the slow-worm *Anguis fragilis* where ACOs were used to capture these habitat generalist legless lizards that are thought to be common and widespread throughout Europe (see [24,25] for descriptions of the natural history of the species). The species is rarely active in the open, and can be hard to detect despite its apparently high abundance [26]. For example, Stumpel [27] captured just one individual in 9.9 h. Even though the species is not classified as threatened, it is protected by law in many European countries and is considered a species of conservation concern. In the UK, for example, translocation of slow-worms is very common before sites can be developed on (if alternative conservation actions are not possible). It is yet unclear how many slow-worms have to be captured and translocated so that one can safely assume that a population was removed and protected or that it has successfully established at a translocation site [28]. For the slow-worm, and many other species that are difficult to observe and are of conservation interest, the development of standardized methodologies for estimating population state variables may help settle the debate on how much effort is necessary to deplete a population such that inadvertent mortality can be avoided as required by the law [28]. Völkl & Alfermann [24] argued that one of the main problems with density estimation of the slow-worm was the lack of a clear definition of space.

Using mark–recapture data that were collected using ACO arrays, we report estimates of density, detection probability, and home range size of slow-worms and we show that these estimates vary geographically. Because ACOs are widely used in herpetology, we believe that SCR methods may generally be useful for the study of the ecology of amphibians and reptiles [18,20].

## 2. Material and methods

### 2.1. Study area

**Figure 1.**
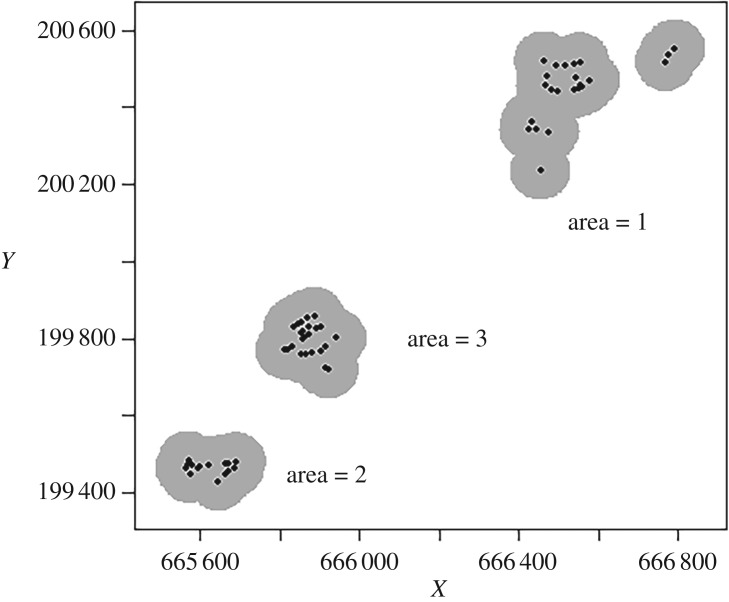
Overview of the three areas (i.e. ACO grids).

We estimated density of slow-worms (*Anguis fragilis*) at Mueterschwanderberg, canton Nidwalden, Switzerland, at 770 m a.s.l. The area is used for extensive agriculture, mostly pastures for cattle grazing. After consulting the local farmers, we selected three areas where the habitats were expected to be most suitable for slow-worms. There were no obvious differences between the three areas. At the three sites, 15, 24 and 23 ACOs made of unalloyed steel and measuring 400 mm × 500 mm × 1 mm, were placed in irregular arrays (figure 1) along hedges or dry stone walls, i.e. preferred slow-worm microhabitat. The mean distance to nearest ACO in the three sites, which was determined by the distribution of suitable slow-worm habitat, was 12.43, 12.76 and 22.81 m respectively. For additional details regarding site selection and ACO placement, see [29].

### 2.2. Data collection

ACOs were checked every 2 days in the morning from 23 April 2012 to the end of July 2012 resulting in 59 checks per ACO. The sequence in which ACO were checked was changed regularly. When an ACO was checked, slow-worms were captured by hand and photographed for later identification. Photographic mark–recapture can be used to distinguish individuals [24,28,30]. Some slow-worms escaped during the capture event and thus detection was not recorded.

### 2.3. Statistical analysis

ACO surveys produce individual spatial encounter histories which we denote by the binary variable *y_ijk_* where *y_ijk_* = 1 if individual *i* was captured under cover object *j* during sample occasion *k*. Of note, the location of capture (the known location of trap *j*) is retained, unlike an ordinary capture–recapture analysis that is based only on the non-spatial ‘individual × occasion’ capture history.

One class of SCR model assumes that individual encounters are Bernoulli random variables with individual- and trap-specific detection probabilities *p_ij_* that depend on the trap (ACO) location *x_j_* and activity centre of individual *i*, *s_i_.* Detection probability is modelled as a decreasing function of *d*(*x_j_, s_i_*), the distance between *s_i_* and *x_j_*. A typical detection function is the half-normal model:

$$p_{ij}=p_{0}\exp\left(\frac{-d{\left(x_{j},s_{i}\right)}^{2}}{2\sigma^{2}}\right).$$

Here *p*_0_, the detection probability of an individual under a cover object located precisely at its activity centre (distance = 0), and *σ*, the spatial scale parameter controlling the decrease in detectability with increasing distance, are parameters to be estimated. A large number of extensions of this basic model are possible by modelling the parameters *p*_0_ and *σ* as functions of individual, trap, or occasion specific covariates. Effects on *p*_0_ can easily be modelled in SCR using a logistic-linear model. For example, studies that occur over a season or over multiple years might have temporal variability in the parameter *p*_0_, such as a linear (or other polynomial) trend (see [20]):

$$\text{logit}(p_{0,k})=\alpha_{0}+\alpha_{1}\;{\text{time}}_{k}.$$

A basic assumption of the model just described is that observations of individuals in traps are independent random variables, and the Bernoulli encounter model allows individuals to be encountered in multiple traps during an occasion. While this is sensible for sampling based on camera traps or ‘hair snares’ (for mammalian carnivores), for population sampling based on ACOs and certain other types of methods, including mist nets in bird studies, encounters of individuals in traps are not independent. This is because an individual can only be encountered in one trap during an occasion because the individual's physical presence is necessary for capture or recapture. In such instances, the encounter model is assumed to be a multinomial outcome with trap-specific encounter probabilities defined by (see [6]):

$$\pi_{ij}=\frac{p_{0}\exp(-d{\left(x_{j},s_{i}\right)}^{2}\text{/}2\sigma^{2})}{1+p_{0}\sum_{j}\exp(-d{\left(x_{j},s_{i}\right)}^{2}/2\sigma^{2})},$$

and the probability of going uncaptured during an occasion is

$$\pi_{i,0}=\frac{1}{1+\sum_{j}p_{0}\exp(d{\left(x_{j},s_{i}\right)}^{2}/2\sigma^{2})}.$$

This relationship between distance *d*(*x_j_*, *s_i_*) and encounter probabilities *π_ij_* is simply the multinomial logit transform for multiple discrete outcomes. The multinomial model for encounters is typically referred to as a ‘multi-catch’ device [31].

SCR models can be thought of as a type of generalized linear mixed model with a random effect (the activity centre *s_i_*), and so they are amenable to standard methods for inference from random effects models using Bayesian [14] or classical methods based on marginal likelihood [13]. We adopt a likelihood analysis of the models here using the R package oSCR (‘oscar’ [32]). The state-space, an area encompassing all plausible activity centres of the observed individuals, was defined by a 3 m regular grid defined by a rectangle enclosing the trap array plus a buffer of 75 m. The oSCR package allows for modelling variation in (or sharing of) parameters among independent strata (called ‘sessions’) which can be defined by multiple trapping grids, as is the case in the slow-worm study, or different temporal periods, or other stratification variables.

We fit a suite of models to the slow-worm ACO survey data including (see also table 1):

(1) the null model having constant detection probabilities and density among the three areas (i.e. grids);
(2) area-specific baseline detection probability per sampling occasion and constant density;
(3) area-specific density and constant detection parameters;
(4) area-specific baseline detection and density;
(5) model 4 but with detection probability (*p*_0_) varying linearly over the sampling period (‘day of sampling effect’);
(6) same as model 5 but a quadratic day of sampling effect;
(7) area-specific density and linear trend in detection (constant baseline detection probability);
(8) same as model 7 but with a quadratic trend in detection.

**Table 1.** Basic parameter estimates, AIC values (lower is better; AIC of the best model = 3051.451) and ΔAIC (difference compared to the ‘best’ model) for the 8 models fitted to the slow-worm data, ordered by AIC (best model in row 1). D(k) is the estimated density (per ha) of slow-worms in area k, $\hat{\sigma }$ is the estimated SCR spatial scale parameter in metres. (.) means that no covariate was used to model the parameter, doy; day of sampling. The parameter estimates for detection probability are presented in the columns ‘intercept’, ‘session(2)’, ‘session(3)’, ‘doy’ and ‘doy^2^’. In all models, sigma was held constant across sessions.

	encounter model					density model				
	intercept	session (2)	session (3)	doy	doy^2^	D(1)	D(2)	D(3)	$\hat{\sigma }$	ΔAIC
D(∼session) p(∼session + doy + doy^2^)	−2.37	−1.19	−1.94	−0.01	−0.01	13.087	45.711	46.979	14.244	0.000
D(∼session) p(∼session + doy)	−2.45	−1.19	−1.94	−0.03	—	13.086	45.742	46.982	14.243	0.057
D(∼session) p(∼session)	−2.51	−1.18	−1.93	—	—	13.108	45.696	46.97	14.25	1.497
D(.) p(∼session)	−2.92	−0.55	−1.23	—	—	27.34	27.34	27.34	14.109	42.56
D(∼session) p(∼doy + doy^2^)	−3.41	—	—	−0.01	−0.01	19.966	45.549	32.894	13.72	55.824
D(∼session) p(∼doy)	−3.50	—	—	—	—	19.97	45.555	32.898	13.721	55.911
D(∼session) p(.)	−3.55	—	—	—	—	19.975	45.564	32.904	13.721	57.056
D(.) p(.)	−3.57	—	—	—	—	30.377	30.377	30.377	13.693	69.474

Note that for all models we assume the spatial scale parameter (*σ*) is constant across areas.

We also calculated densities based on the number of unique individuals encountered during the study. In order to express the number as a density, we had to calculate the area of the sessions. While this area is well defined when using SCR models, it requires assumptions regarding the effective trapping area [6–8]. To calculate the area, we created a buffer around the ACO array using the mean maximum distance moved (MMDM [6]) [20]. We calculated the areas using buffers based on both the full MMDM and 0.5*MMDM [20].

## 3. Results

The number of captured individuals in each area (i.e. cover board grid; ‘session’) was 44, 50 and 57 for areas 1, 2 and 3 respectively. The three trapping areas and the defined state space (grey rectangles) are shown in figure 1. The average number of captures per area was 2.55, 2.20 and 1.58, respectively. The mean maximum distance moved (MMDM) varied among area (68.81, 12.18 and 28.99 m, respectively; not including movement distances which were zero).

**Figure 2.**
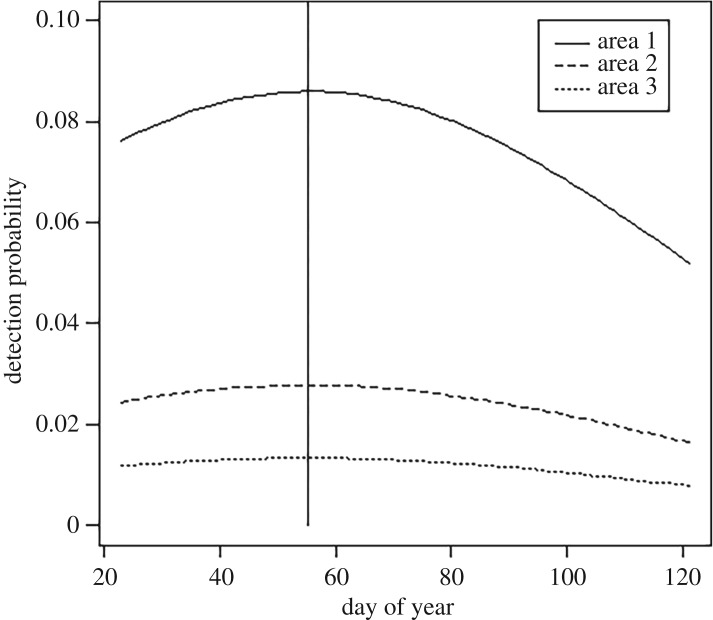
Estimated quadratic day of sampling effect on probability of detection at distance 0 from an individual home range centre.

Model selection using AIC showed that detection was best modelled using an effect of the day of sampling; specifically a quadratic effect of day of sampling on slow-worm encounter probability. The estimated effect is shown graphically in figure 2. The quadratic effect indicates a distinctive maximum probability of detection approximately 55 days into the sampling season (16 June, table 1). There is strong variation among areas in the baseline probability of detection which is 0.086 (s.e. 0.0148), 0.028 (s.e. 0.0045) and 0.013 (s.e. 0.0019) for areas 1, 2 and 3 respectively, i.e. slow-worm encounter probability, as well as being higher in the middle of the sampling period, is also higher on any day of sampling in area 1 than in areas 2 or 3, which are similar (figure 2).

The best models all suggest a high degree of variation in density among the three spatial sampling areas, with area 1 in the northeast corner of the study area having the lowest estimated density (13.10 slow-worms ha^−1^, s.e. 1.97), and areas two and three having higher densities, 45.71 (s.e. 6.46) and 46.98 (s.e. 6.22) slow-worms ha^−1^, respectively. The estimated spatial scale parameter is *σ* = 14.25 which, under a bivariate normal model, suggests a 95% space use area of π*5.99*(14.25^2^) = 3821 m^2^ or 0.38 ha (s.e. = 429 m^2^).

Using MMDM to calculate the area of the session, we estimated densities of 4.6, 26.6 and 20.2 individuals per hectare, respectively. Using 0.5*MMDM, the densities were 6.0, 45.8 and 30.7 individuals per hectare, respectively.

## 4. Discussion

Abundance is of central interest to both ecologists and conservation biologists. Here, we provided density estimates for a species for which the lack of reliable information about density has hindered conservation action [28]. Völkl & Alfermann [24] argued that one of the main problems with density estimation of the slow-worm was the lack of a clear definition of space. Here, using spatial capture–recapture methods that incorporate space explicitly in the model, we estimate slow-worm density free from such criticisms.

The density estimates were well within the range of those considered to be reliable [24]. While we also detected strong spatial variation in density, we agree with Völkl & Alfermann [24] that previous density estimates are probably overestimates. Our estimates of density may be useful for informing translocation of slow-worms to calibrate expectations of how many individuals have to be captured at a site, but also what densities constitute an established population. Spatial variation in density, however, implies that there is no single value that may be applied to all populations. To be conservative, we recommend that the higher densities are used for planning and assessing translocation projects. We do not know the cause for the variation in density among the three areas. It may be related to social interactions, resource availability or habitat quality [33–35]. Further studies that link density to resource selection and or variation in habitat quality may therefore be worthwhile [36]. Moreover, increasing the variety of habitat types and overall area sampled, SCR can be used to directly answer such questions.

The estimated densities of slow-worms based on the number of unique individuals that were encountered during the study were considerably lower than the SCR estimates and they depended strongly on the way we calculated the area of the trapping grids (i.e. sessions). Thus, we argue that the use of SCR methods provides more reliable estimates of density than other approaches (e.g. simple counts).

An interesting product of density estimation using spatial capture–recapture models is the estimate of the spatial extent of slow-worm space use, which can be thought of as analogous to home range size. To the best of our knowledge, this study represents the first estimates of slow-worm home ranges. Under a bivariate normal home range model [6], the estimate of the 95% home range area was 0.38 ha (s.e. = 0.0429 ha). This is substantially larger than the estimate in some management guidelines for reptiles (‘only several hundred square metres’; [37]). Home ranges vary widely among reptile species [38]. Our estimate of slow-worm home range size is well within the range of known reptile home range sizes. Reptile species which are known to occur syntopically with slow-worms, the smooth snake (*Coronella austriaca*) and sand lizard (*Lacerta agilis*), have larger (up to 1.8 ha; [39]) and smaller home ranges (0.06 ha, [34]), respectively, which is consistent with an effect of body size on home range size.

We used an irregular ACO array in which ACOs were placed along hedges and other linear habitat elements (such as dry stone walls) that were identified as suitable habitat for slow-worms. Trap spacing is important because, in SCR, some individuals should be captured under multiple ACOs resulting in unique spatial recaptures. The recommended trap spacing in SCR studies is about 2*σ* [40,41] and was roughly 1 to 1.5*σ* in this study. For the slow-worm, the distance should be roughly 28 m (the exact value may depend on habitat type and heterogeneity). This is almost equivalent to the greatest inter-ACO distance used by Reading [19]. Reading [19] showed that the number of captures per hectare increased with decreasing inter-ACO distance. However, when using the recommended inter-ACO distance of 2*σ* of roughly 28 m, then one may cover a larger area with the same number of ACOs. Thus, the estimate of *σ* may be useful when designing new surveys of the slow-worm and species with similar natural history.

We believe that spatial capture–recapture methods are a very promising method for herpetological field studies [20]. While there is a large number of methods that can be used to estimate abundance, only spatial capture–recapture has a natural way of incorporating space explicitly. This is of fundamental importance since there is no natural spatial unit that could be used to delineate the spatial extent of a reptile population (as there is for pond-breeding amphibians where there is the shortcut ‘pond = population’). As we have shown here, spatial capture methods can also provide an inferential framework for spatial variation and/or gradients in density if gradients are representatively sampled. Thus, density estimates based on spatial capture–recapture methods may allow us to address many questions in basic and applied spatial ecology.

## Data accessibility

Data and R code are available on Github (https://github.com/jaroyle/slowworm).
